# Clearing the Air, Breathe Easy: Intensive Care Unit Remodeling Unveils Insights into Aspergillosis Prevention

**DOI:** 10.1017/ash.2024.287

**Published:** 2024-09-16

**Authors:** Kinta Alexander, Sean Brown, Charlotte Ozuna, Kim Moi Wong Lama

**Affiliations:** New York City Health + Hospitals; Harlem Hospital

## Abstract

**Background:** Invasive aspergillosis (IA) poses a substantial threat to morbidity and mortality, particularly among immunocompromised individuals. In 2023, a New York City Intensive Care Unit (ICU) experienced an aspergillus outbreak following a structural water leak, resulting in two patients diagnosed with Invasive Aspergillus niger in their bronchial cultures. Immediate interventions, including patient relocation and ICU reconstruction were implemented to mitigate further impacts. This study aims to assess the impact of timely relocation of patients and renovation of the ICU, on the incidence of invasive aspergillosis. **Method:** A quasi-experimental study design of ICU patients over a nine month period included surveillance by the Infection Prevention department from March 1 to December 1, 2023. Surveillance included review of microbiology reports, environmental cultures, and patient chart reviews. The Pre-intervention spanned March 1 to May 1, and the post-intervention from May 4 to December 1. Indoor mold assessments of pre- and post-intervention involved testing wall surfaces for moisture, air sample collection for fungal spores, and surface swabs for direct fungal analysis. The intervention included relocating all seventeen patients from the impacted ICU and comprehensive reconstruction. Reconstruction involved the removal and replacement of all sheetrock within the unit extending four feet from the floor with moisture-resistant sheetrock. Additionally, moisture resistant single sheet welded vinyl flooring and cove-bases were installed. All heating, ventilation, and air-conditioning (HVAC) systems were inspected and cleaned. Construction activities strictly adhered to Infection Control Risk Assessment (ICRA) guidelines, with emphasis on maintaining negative pressure, to ensure a safe environment. **Result:** Environmental swab samples from 50% of ICU rooms indicated growth of Aspergillus/Penicillium, Chaetomium, and Stachybotrys/Memnoniella type spores during the pre-intervention phase. Environmental microbiology results strongly suggest the indoor environment as the fungal spore source, with the presence of fruiting structures indicating surface mold growth. Indoor air samples, when compared to outdoor samples collected during pre-intervention, showed rare (2-6 raw count) growth of Aspergillus in 55% of the sampled rooms and subequently no growth post-intervention. Prospective surveillance revealed no further aspergillus growth in the ICU population and environment. **Conclusion:** Our findings highlight a potential correlation between environmental modifications and reduced IA incidence. Swift mitigation and structural interventions are crucial in averting potentially fatal outcomes, marking a significant advancement of prevention strategies for inner-city hospital settings. Although promising, study limitations include the inability to speciate environmental aspergillus for comparison to patient bronchial cultures and the absence of baseline bronchial cultures for affected patients on admission.

